# Trait Anxiety and Unhealthy Eating in Adult Women: The Mediating Role of Cognitive Instability

**DOI:** 10.3390/ijerph20010296

**Published:** 2022-12-24

**Authors:** Magdalena Mróz, James J. Gross, Anna Brytek-Matera

**Affiliations:** 1 Institute of Psychology, University of Wrocław, 50-527 Wrocław, Poland; 2 Department of Psychology, Stanford University, Stanford, CA 94305, USA; 3 Katowice Faculty of Psychology, SWPS University of Social Sciences and Humanities, 40-326 Katowice, Poland

**Keywords:** cognitive instability, trait anxiety, unhealthy eating, women

## Abstract

Previous studies have demonstrated the influence of affective states on unhealthy eating. Heightened impulsivity has also been recognized as a risk factor for unhealthy eating. The objective of the present study was to investigate the relationship between trait anxiety and unhealthy eating and to test whether cognitive instability (trait impulsivity component) might mediate this relationship among adult women. Method: The sample was composed of 225 women (*M*_age_ = 29.70 ± 9.23; *M*_BodyMass Index_ = 23.39 ± 4.43). The State-Trait Anxiety Inventory (STAI), the Food Frequency Questionnaire (FFQ), and the Barratt Impulsiveness Scale (BIS) were used. We first conducted a principal components analysis to reduce the dimensionality of the FFQ data, finding four principal components. For our primary analyses, we focused on unhealthy eating. We then conducted a mediation analysis to examine whether trait anxiety was associated with unhealthy eating and, if so, whether cognitive impulsivity mediated this relationship. Results: Trait anxiety was positively associated with cognitive instability, and cognitive instability was positively associated with unhealthy eating. Trait anxiety was also positively associated with unhealthy eating, but only when controlling for cognitive instability. Consistent with our proposed mediation model, trait anxiety was positively associated with cognitive instability, which in turn predicted unhealthy eating among adult women. Conclusion: Adult women who reported higher levels of trait anxiety experienced higher levels of cognitive instability and engaged in poor dietary behaviors. Identifying the factors related to unhealthy eating represents a crucial step toward findings ways of reducing their impact on food intake and replacing them with more productive ones.

## 1. Introduction

Emotional distress leads to greater consumption of unhealthy foods (e.g., processed foods, junk food, and snacks, especially desserts, chocolate, ice creams, and salty snacks) [1]. This signals a rise in unhealthy eating, which may be defined as diet patterns inconsistent with generally accepted principles of healthy eating (including intake of vegetables, fruit, whole grains, fish, legumes, and unprocessed meat), and which generally includes a higher intake of sugary, processed foods, fatty foods (including sweets, fast food, and snacks) [2]. This is concerning because unhealthy eating promotes pro-inflammatory processes, which cause a deterioration in health (e.g., higher risk of cardiovascular disease or diabetes) and reduced cognitive ability [3,4,5].

### 1.1. Emotional State and Unhealthy Eating

An extensive body of literature, e.g., [6,7,8] has linked affective states to the onset and maintenance of unhealthy eating. However, the precise impact of affective states on unhealthy eating is inconclusive. For example, Macht [9] indicates that intense emotions or strong tension can lead to the suppression of eating; however, depending on the motivation for eating, intense emotions or those triggered by moderate tension can lead to an overall increase in food intake or to an increase in the intake of sweet and fatty foods [9].

Additionally, emotional valence (positive or negative emotional state) affects eating behavior inconsistently, as seen in changes in the amount of food intake and unhealthy food choices in response to a positive or negative mood [6,10,11]. The associations between negative mood and greater food intake in healthy controls and clinical samples have been observed in the meta-analysis [10]. In the same study, another comparison between positive and neutral mood demonstrated that positive mood was associated with greater caloric intake only in healthy controls [10]. Another meta-analysis did not confirm those findings. Only positive emotions had a small effect on greater food intake [11]. Furthermore, Devonport et al. [6], in a meta-analysis, showed that food intake increased under the influence of positive affect compared to negative (comparison between positive and negative mood). However, studies that focused on the impact of negative emotions on eating behavior have shown that food is used to regulate emotional distress for people, both normal-weight and overweight, which leads to unhealthy eating behavior and poor food choices in response to negative emotions [6]. 

The results of a recent scoping review, including 1541 articles [2], suggest a possible association between higher levels of anxiety and poor dietary patterns (e.g., high-fat diet, high intake of sugar and refined carbohydrates, artificial sweeteners, unhealthy eating, snacking). Further complicating the picture, it appears that there may also be gender differences in the effects of affect on eating. Recent studies have indicated that women with high levels of trait anxiety consume more added simple sugars compared to those with low trait anxiety [12], but this effect was not observed in men [13]. 

Although the influence of emotional valence on eating behavior varies, increasing food intake and more frequent consumption of unhealthy food are observed as regulating emotional strategies, especially negative ones (such as anxiety). This is in line with the results of a recent study [1], which found that emotional distress seems to be the primary mechanism explaining an increase in comfort food intake during the crisis. This leads us to the conclusion that negative affective states can promote unhealthy food intake.

### 1.2. Impulsivity and Unhealthy Eating

Impulsivity is defined as a tendency to unplanned, rash action without careful thinking [14]. Impulsive action is performed without regard for possible immediate and long-term consequences [14], and it has been speculated that impulsivity may be linked to unhealthy eating [15,16]. Impulsive tendencies may drive food choices, and some individuals may find it more difficult to resist impulses related to unhealthy food choices. Moreover, low self-control and high impulsivity have been suggested to be associated with unhealthy behaviors [17]. In addition, it has been suggested that the traits of self-control and impulsivity lie at the root of the varying degrees of ability to resist impulses to consume more palatable, energy-dense foods and stop oneself from giving in to unhealthy food choices [18]. 

The gold standard in measuring impulsivity as a trait is the Barratt Impulsiveness Scale Version 11 (BIS-11) [19]. Originally, the BIS-11 was developed as a multi-faceted measure of personality [19]. However, the factor construct of the BIS-11 has recently been questioned as not having empirical support [20]. Consequently, we did not use the BIS-11 to assess the global level of impulsivity. We focused on evaluating a single subscale (cognitive instability), which corresponds to the common emotional coping style of worrying and ruminating among women [21]. These strategies are possible reasons for sex differences: women experience more anxiety than men [22]. The selected subscale appears to be the best method to assess the postulated relationship. 

A recent study [23] found that higher scores on the BIS-11 subscale “attentional impulsiveness” were observed in normal-weight individuals (at risk for binge eating disorder). “Attentional impulsiveness” has been shown to be associated with binge eating in both normal-weight women [24] and those with obesity [25]. In summary, heightened scores on the “attentional impulsiveness” subscale indicate a risk of unhealthy eating behaviours leading to obesity or eating disorders. 

For clarity, we refer to studies that reported scores of the “attentional impulsiveness” subscale (consisting of two subscales: cognitive instability and attention) or the BIS-11 total score because previous studies have rarely reported results of first-order subscales such as cognitive instability [20]. A detailed description of BIS-11 and the cognitive instability subscale is described in Section 2.2.3.

### 1.3. The Objective of the Present Study: Impulsivity, Anxiety, and Unhealthy Eating

To the best of our knowledge, the mediating role of cognitive instability between trait anxiety and unhealthy eating behavior has not been assessed to date. However, impulsivity was successfully applied as a moderator of the relationship between social anxiety and another maladaptive emotion regulation strategy [26].

In addition, studies have shown associations of negative emotions (including anxiety) with increased impulsivity and a tendency to unhealthy eating behavior [27,28,29]. Women diagnosed with a generalized anxiety disorder have demonstrated that higher levels of impulsivity predict higher saturated fat and sugar intake [27]. More generally, young female adults with a normal body mass index (BMI) overate most often under the influence of anxiety (51.3%). In addition, they used periodic episodes of overeating as a coping strategy for negative emotional states, which were positively associated with eating disorder symptoms and loss of control over food intake [28]. Another study revealed that it was mostly women with a higher BMI who tended to be more impulsive and eat more in response to negative emotions [29]. 

To sum up, the effects of heightened impulsivity associated with increased anxiety can lead to unhealthy eating behaviors among healthy adult women, which can result in the development of obesity and/or eating disorders [30]. In the rising number of studies reporting positive associations of anxiety and impulsivity and unhealthy eating, we were interested in investigating whether the trait of impulsivity (understood as intrusive thoughts) can more precisely explain the anxiety-induced tendency to consume unhealthy food. 

In this study, we predicted that trait anxiety would be positively associated with self-reported unhealthy eating and that cognitive instability would mediate this relationship such that participants who reported higher levels of trait anxiety would experience higher levels of cognitive instability, and cognitive instability would be positively associated with unhealthy eating. Given prior evidence of gender differences, our focus was on adult women, who seem most prone to anxiety-related unhealthy eating.

Developing knowledge of the factors influencing the relationship between anxiety and eating behaviors will contribute to a better understanding of the relationship between emotions and eating behaviors and the personality factors (impulsivity) that influence them. This will enable the planning and creation of more effective forms of support for women experiencing anxiety and the design of prevention programs aimed at developing healthy eating habits in a group of healthy individuals with normal weight [31].

## 2. Methods

### 2.1. Participants and Procedure

A total of 225 Polish adult women participated in the present study. The mean age of participants was 29.70 years (SD = 9.23). The mean BMI was 23.39 kg/m^2^ (SD = 4.43) indicating healthy weight range [32] (Table 1).

The participants were recruited through university centers, health clubs, fitness gyms, companies as well as through social media. Participants were given written information about the study and were asked to sign a consent form. All participants offered their informed consent before starting the survey (by marking a respective box at the first page of the survey) and responded voluntarily to the survey. Women were informed about the anonymity of the study, and about the possibility of resignation at any stage of the study.

The inclusion/exclusion criteria included the following characteristics: (1) women ≥ 18 years of age; (2) non-vegetarian and tries to eat healthily on a regular basis, but also enjoys eating junk food and snacks; (3) not allergic to major groups of food (e.g., gluten allergy); (4) no current or past-year history of major depression (which can affect appetite and weight) or one of three major eating disorders (anorexia nervosa, bulimia nervosa, binge eating disorder); (5) no abuse of drugs or alcohol (which can affect appetite and weight); (6) and no lifetime history of psychosis, mania, hypomania, bipolar disorders, or suicidality, defined using the Mini International Neuropsychiatric Interview (MINI) [33]. The electronic version of the MINI was used through the Nview Health portal (portal.nviewhealth.com). Only individuals with a low total score on the interview sections were invited to participate in the present study. All data except those for trait anxiety were collected through Survey Monkey^®^ (San Mateo, CA, USA). For assessing trait anxiety, we used the paper-and-pencil assessment. The response rate was 85%. Data from 37 participants (16.44%) were removed due to missing data exceeding 30%.

The current study is a part of the Harmonia 10 research project funded by the National Science Centre (Poland; grant no. 2018/30/M/HS6/00022) on the impact of negative affect on eating behavior in ecological and natural settings. The theoretical framework of the project as well as the procedure have been presented in the previous publications [34,35]. The research protocol of the Harmonia 10 research project was approved by the Research Ethics Committee at the Institute of Psychology, University of Wroclaw, Poland (decision number IPE 0019), and was designed and conducted in accordance with the guidelines of the Declaration of Helsinki.

### 2.2. Measures

#### 2.2.1. Trait Anxiety

Trait anxiety was assessed using the State-Trait Anxiety Inventory (STAI) [36]. In the present study, we used the Polish version of the STAI [37]. The STAI is one of the most common self-report anxiety assessment tools. It consists of two independent questionnaires that measure state and trait anxiety. Each subscale of the STAI consists of 20 items. In the present study, we assessed trait anxiety, which is defined as a relatively permanent personality trait. Items describe tendencies to perceive stressful situations as threatening and more stable aspects of anxiety proneness, e.g., “I worry too much over something that really doesn’t matter”. Respondents answered on a 4-point Likert scale (from 1 = almost never to 4 = almost always). The score ranges from 20 to 80 points. The higher the scores are, the greater the proneness to anxiety is. Internal consistency for the scale (Cronbach’s alfa) was 0.88 in the present study. 

#### 2.2.2. Unhealthy Eating (Eating Behaviors)

Unhealthy eating was assessed using the Food Frequency Questionnaire (FFQ). The Polish version of the FFQ [38] consists of a list of foods and beverages with response categories to indicate usual frequency of consumption over the time period queried. To assess the total diet, the number of foods and beverages queried typically ranges from 80 to 120 (National Cancer Institute) [39]. In the present study, adult women were presented with twenty-one questions about eating behaviors over the preceding two months (Table 2). Participants responded on an 8-point scale (from 1 = several times a day to 8 = never). As described in Section 3.2 we used principal components analysis (PCA) to reduce the dimensionality of the FFQ data.

We opted to use this measure over a 24-h dietary recall because we were interested in collecting detailed information on foods and beverages consumed over a longer period. We opted to use these questions over the FFQ as we wanted a measure that women could complete in a few minutes or less.

#### 2.2.3. Cognitive Impulsiveness Component: Cognitive Instability

Cognitive instability was assessed using the Barratt Impulsiveness Scale Version 11 (BIS-11) [40]. The BIS-11 is one of the most widely used measures to assess self-reported impulsivity. The current version of the instrument was revised by Patton et al. [40] and reconsidered by Stanford et al. [19]. The Cronbach’s α for the BIS-11 was α = 0.83 [19]. We used Polish BIS-11 [41]. The BIS-11 contains 30 items, which are divided into six scales (first-order factors): (1) attention (consists of 5 items, e.g., I concentrate easily.); (2) cognitive instability (3 items, e.g., I have “racing” thoughts); (3) motor impulsiveness (7 items, e.g., I make-up my mind quickly); (4) perseverance (4 items, e.g., I change jobs); (5) cognitive complexity (5 items, e.g., I like puzzles); (6) self-control (6 items, e.g., I plan tasks carefully). Items are rated on a 4-point Likert scale (from 1 = rarely/never to 4 = almost always/always). 

Originally, Barratt developed a one-dimensional scale to assess impulsivity [42]. Furthermore, based on psychometric analysis, Patton et al. [40] made the 11th revision of the BIS-11 scale, in which the principal components analysis showed six primary factors (the six scales of BIS-11 mentioned above). Subsequent investigations revealed the possibility of creating three second-order factors (scales) that corresponded to Barratt’s theory [40]. The second-order scales consist of (1) “attentional impulsiveness” (including cognitive instability and attention scales), (2) motor impulsiveness (including motor impulsiveness and perseverance scales), and (3) non-planning impulsiveness (including self-control and cognitive complexity scales) [20]. According to Patton et al. [40], impulsivity is a multi-faceted construct reflected in the BIS-11 factor (subscales) structure, and it is recommended to report the first-order subscales results [40]. Therefore, in the current study, we focused on one subscale, cognitive instability, which describes impulsivity as a propensity for intrusive thoughts. The Cronbach’s α for the cognitive instability subscale was 0.58 in our study. Cronbach’s α calculated on the adult sample (N = 1577) has shown similar internal consistency α = 0.55 [19].

### 2.3. Statistical Analysis

Data were analyzed by using IBM SPSS Statistics for Windows, Version 26.0 (IBM Corp., Armonk, NY, USA). We first conducted a PCA to reduce the dimensionality of the FFQ data. Then, we measured the relationship between variables using Pearson correlation coefficient (r). Finally, the PROCESS macro [43], model 4, version 4.0 in SPSS with bias-corrected 95% confidence intervals (CIs) with 10,000 bootstrapped samples was used to test the hypothesized mediation models between trait anxiety, cognitive instability, and unhealthy eating. The regression analyses were performed for each path, regressing the predictor (trait anxiety) on the outcome (unhealthy eating) and the mediator (cognitive instability). 

Taking into consideration the current understanding of mediation testing [43], in the present study, path *a* represents the relationship between trait anxiety and cognitive instability. Path *b* represents the relationship between cognitive instability and unhealthy eating when controlling for trait anxiety. Path *c* represents the relationship between trait anxiety and unhealthy eating (total effect). Path *c’* represents the relationship between trait anxiety and unhealthy eating when controlling for cognitive instability (direct effect). In addition, the indirect effect (i.e., mediation) is the product of the *a* and *b* path.

## 3. Results

### 3.1. Preliminary analyses

The main characteristics of adult women are presented in Table 3.

The descriptive characteristics of the participants are presented in Table 4.

We conducted a PCA on twenty-one questions about eating behaviors over the preceding two months (Table 3). We used PCA to reduce the dimensionality of the data to a small number of components (Table 5). Similar to the recent study [44], instead of examining twenty-one separate sets of models (i.e., one for each item), we investigated a limited number of regression and mediation models. Based on the Kaiser-Guttman rule [45], we kept all the components with eigenvalues greater than 1.

Using varimax rotation, we extracted four principal components from the FFQ: healthy eating (the first principal component), consumption of gluten-free and organic food products (the second principal component), unhealthy eating (the third principal component), and water (the fourth principal component) (Table 6).

For our primary analyses, we solely focused on the unhealthy eating component (i.e., sweet and unhealthy foods, snack foods).

### 3.2. Correlation Analysis

Prior to the mediational analyses, Pearson correlation coefficients were calculated to examine associations of study variables. In our population, trait anxiety was associated with unhealthy eating (r = 0.155, *p* = 0.020) and cognitive instability (r = 0.624, *p* = 0.001). A relationship between cognitive instability and unhealthy eating (r = 0.208. *p* = 0.002) was also found.

### 3.3. Mediation Analysis

We conducted a mediation analysis for examining whether the trait anxiety was associated with unhealthy eating and for assessing whether cognitive impulsivity mediated this relationship.

**Figure 1:**
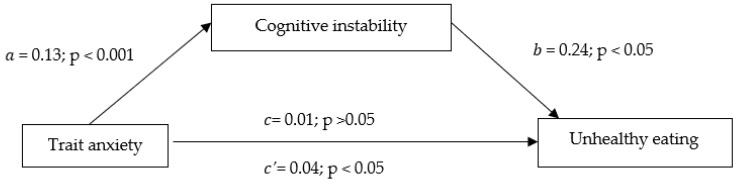
Mediation model for the effect of cognitive instability in the relationship between trait anxiety and unhealthy eating in adult women.

The results indicated that the path *a* model was significant (R^2^ = 0.41, F(1,223) = 156.15, *p* < 0.001), showing that higher levels of trait anxiety significantly predicted higher levels of cognitive instability. Path *b* was also significant (R^2^ = 0.04, F(2,222) = 5.11, *p* = 0.006), indicating that higher levels of cognitive instability significantly predicted more frequent unhealthy eating. Path *c* was not significant (R^2^ = 0.22, F(2,185) = 26.97, *p* = 0.660), demonstrating that trait anxiety was not associated with unhealthy eating (95% CI [−0.05, 0.03], *p* = 0.667). However, path *c’* was significant, demonstrating that greater trait anxiety significantly predicted more frequent uncontrolled eating when controlling for cognitive instability (95% CI [−0.08, −0.00], *p* = 0.019) (Figure 1 and Table 7).

The indirect effects and total effects were significant, while the direct effect was not significant. Thus, the full mediation was presented (the direct effect was not significant but the indirect effect was significant).

## 4. Discussion

The objective of the present study was to investigate the relationship between trait anxiety and unhealthy eating and the mediating effect that cognitive instability (trait impulsivity) could have in this relationship among adult women of normal weight. 

### 4.1. Relationship between Trait Anxiety and Cognitive Instability: Path a

We found that trait anxiety was positively related to cognitive instability. It means that trait anxiety was associated with a higher severity of intrusive thoughts. Our results are consistent with previous studies demonstrating that anxiety was the strongest predictor of the total BIS-11 score and “attentional impulsiveness” subscale in young (18–30 years) and middle-aged (31–49 years) individuals [46]. To date, no study has been reported on the relationship between cognitive instability and anxiety among a non-clinical group, thus we refer to the more complex BIS-11 results.

However, studies conducted in a clinical sample have indicated that the scores of the BIS-11 cognitive instability and “attentional impulsiveness” subscales are convergent: individuals with anorexia nervosa had significantly higher scores in both subscales compared to healthy controls [47]. Regardless of the severity of eating disorder symptoms, anxiety, depression, and stress were found to be positively associated with self-reported impulsiveness [47]. Studies conducted among non-clinical and clinical samples have supported the relationship between anxiety and impulsivity. These data allow us to assume that the results we obtained are reasonable.

### 4.2. Relationship between Cognitive Instability and Unhealthy Eating: Path b

The present study focused on the assessment of the relationship between the primary factor of impulsivity, cognitive instability, and unhealthy eating in a non-clinical sample. We found a positive relationship between high impulsivity with snacking and eating sweet and unhealthy foods. These findings are consistent with those [15] investigating the relationship between impulsivity and food intake (N = 35,830), snacking (N = 48,562), and eating disorders (N = 48,824) in a general population. Higher impulsivity was associated with a higher intake of snacks and a lower intake of fruit, vegetables, meat, poultry, processed meat, dairy products, and milk-based desserts [15]. Similarly, another study has shown that increased impulsivity exacerbated the choice of unhealthy food among healthy individuals. Increased impulsivity and inhibition deficits were also associated with increased emotional eating, overeating in response to external cues, and making food decisions without considering health values [18]. Another study demonstrated that induced impulsiveness in a normal-weight individual led to a rise in caloric intake [48], which is in line with the positive relationship between cognitive instability (impulsiveness trait) and unhealthy eating observed in our study.

Increased impulsivity, both measured as a personality trait and laboratory-induced, exacerbates unhealthy eating behavior. Individuals characterized by increased impulsivity consume more caloric foods (e.g., sweet, fatty), eat under the influence of experienced emotions, and have greater difficulty in self-controlling their behavior. In addition, the current study indicates that the propensity for intrusive thoughts plays a significant role in the severity of unhealthy eating behavior among women.

### 4.3. Relationship between Trait Anxiety and Unhealthy Eating: Path c

In the present study, trait anxiety was not associated with unhealthy eating among adult women with normal weight. In contrast, the previous study [49] demonstrated that higher trait anxiety was strongly associated with a greater consumption of high-fat foods, higher caloric intake, and higher BMI among women. Notably, women with a high level of emotional eating consume more comfort food, while women with a low level of emotional eating tend to eat less under stress [50]. The literature indicates that the increased consumption of unhealthy food under the influence of anxiety, depression [51], and stress [52] might be considered as coping strategy for negative emotions and negative affect [53]. 

In conclusion, the lack of an association between anxiety and unhealthy food in our study is an expected result. The lack of confirmatory references may be due to the less frequent publication of studies with insignificant results. Since the literature indicates that eating unhealthy food is a maladaptive strategy for emotion regulation, and the findings are inconsistent, it is reasonable to look for factors that mediate the relationship between trait anxiety and unhealthy eating.

### 4.4. The Mediating Role of Cognitive Impulsiveness in the Relationship between Trait Anxiety and Unhealthy Eating in Adult Women Sample: Path c’

The results supported the hypothesis that cognitive instability mediates the association between anxiety as a trait and unhealthy eating. Women who reported higher levels of trait anxiety experienced higher levels of cognitive instability and subsequently engaged in poor dietary behaviors. To the best of our knowledge, this is the first study investigating the mediating role of cognitive instability between anxiety as a trait and unhealthy eating. Therefore, we cannot interpret the results of the current study with other studies based on the BIS-11. However, a laboratory study showed that greater impulsivity (measured with the Impulse subscale from the Difficulties in Emotion Regulation Scale) was associated with higher calorie intake in a stressful situation. In addition, emotional control and impulsivity moderated the relationship between negative affect, food choice, and calorie intake under stressful conditions [54].

Our results are consistent with the model proposed by Macht [9]. Depending on the motivation to eat, intense emotions or moderate emotional tension affect the quantity and quality of food consumed. Emotional eaters regulate their emotions through an increased consumption of sweets and high-fat foods [9]. Furthermore, based on the process model of emotion regulation [55], emotional eating should be treated as a maladaptive emotion regulation strategy, i.e., emotional suppression [55]. Emotional suppression has been found to be associated with higher BMI among adults, mediated by emotional eating and decreased fruit and vegetable consumption [56]. Moreover, our results are consistent with those presented in the recent review [34], indicating two possible pathways for the influence of negative affect on eating behaviors: (1) negative emotions increase the appetite for comfort food and eating causes a reduction in a negative mood and (2) the impaired top-down cognitive control over eating behavior reduces conscious decision making about eating healthy foods.

Our study shows that cognitive instability as a trait of impulsivity may help clarify the direction of the impact of affective states on unhealthy eating behavior. The results indicate that the observation of impulsivity may contribute helpful information in interpreting changes in eating behavior about experienced trait anxiety. This hypothesis needs to be investigated further, but the need to involve executive functions in decision making about eating behavior supports this prediction line. Moreover, further investigation of the role of impulsivity in the development of unhealthy eating in terms of differentiating emotion-related impulsivity and impulsivity as a measure of executive function efficiency is indicated.

Interventions targeting cognitive control are recommended for the prevention of overweight and obesity. Based on a review of 50 articles on cognitive training or neuromodulation among healthy individuals and/or those with excess weight, it was found that response inhibition training is an effective intervention that reduced unhealthy eating and decreased BMI [57]. In addition, programs that promote mindful eating and emotional regulation can be helpful in maintaining a healthy weight [57].

To sum up, our findings can be used in training and prevention programs (emotional states management and nutrition programs). Such programs may help to replace automatic and unreflective tendencies with new healthy behaviors based on training cognitive functions and exercising adaptive strategies for regulating affective states and healthy eating. 

### 4.5. Limitations of the Study and Future Directions

The present study has some limitations. A cross-sectional study cannot provide cause and effect relationships among trait anxiety, unhealthy eating behaviors, and cognitive instability among adult women. There is a risk of response bias because self-reported data were used. Women might offer biased estimates of self-assessed behavior due to misunderstanding some questions and/or social-desirability bias. A low reliability value of the cognitive instability subscale (α = 0.58) was obtained. Finally, the consideration of only one aspect of healthy/unhealthy eating behavior (from the PCA) was observed in the present study. In our study, we focused on assessing impulsivity as a personality trait. In future studies, we recommend expanding impulsivity measurement to include Go/No-Go behavioral testing methods (for a description of inhibition control) [18,58]. 

In future studies, it will be worth investigating the level of reward sensitivity in combination with impulsivity [59]. Individuals with obesity have a stronger preference for an immediate food reward than normal-weight individuals (which is not observed for other rewards: money and discount coupons). In addition, future interventions should address unhealthy food consumption by focusing on handling trait anxiety.

### 4.6. Strengths of the Study

This is the first study to assess the mediating role of cognitive instability between trait anxiety and unhealthy eating. The study is interdisciplinary, combining the fields of psychology and dietetics. Therefore, specialists in both fields can use the results when planning psychological or dietary interventions. Only women participated in the study, which made the sample homogeneous. Women at every stage of their lifespan may be exposed to anxiety that exceeds their coping capacity [22], so the results may be helpful in the prevention of developing anxiety disorders in women. Due to the high prevalence of emotional disorders (anxiety and depression) in the Polish population [60], the study brings valuable information that can help improve Poles’ mental and physical health.

## 5. Conclusions

Our findings demonstrated that greater levels of trait anxiety are related to greater cognitive instability and lead to increased unhealthy eating behaviors. Thus, we can suppose that adult women may consume sugary and snack food due to higher trait anxiety coexisting with higher cognitive instability (intruding thoughts). Identifying the critical impact of intruding thoughts on the relationship between anxiety and unhealthy eating may help change eating patterns and thus contribute to improving both physical and mental health among women.

## Figures and Tables

**Table 1:** Mean, standard deviation, minimum, maximum, skewness, and kurtosis values of age and body mass index for adult women (N = 225).

Measure	Mean	SD	Min	Max	Skewness		Kurtosis	
					Statistic	Std. Error	Statistic	Std. Error
Age	29.70	9.23	20	60	1.06	0.16	0.05	0.32
BMI	23.39	4.43	18.57	42.45	1.45	0.16	3.12	0.32

Note: BMI: body mass index; SD: standard deviation; Min: minimum: Max: maximum; Std. Error: standard error.

**Table 2:** Twenty-one questions about eating behaviors over the preceding two months over the FFQ.

**During the past 2 months, how often have you eaten or drank...**
… low calorie foods?
… fresh foods?
… well-balanced diet?
… low carbohydrate foods?
… organic foods?
… low fat foods?
… natural foods?
… whole grains?
… low sugar foods?
… vegetarian/vegan?
… gluten-free foods?
… low sodium foods?
… locally sourced foods?
… vegetables?
… fruits?
… something sweet and unhealthy (i.e., candy or cake)?
… soft drinks or soda?
… alcoholic beverages?
… snack foods?
… fast foods?
… water?

**Table 3:** Socioeconomic status and eating habits of adult women (N = 225).

Variable	Number	Percent
Body Mass Index range		
Healthy Weight range (18.5 to 24.9 kg/m^2^)	162	72.0
Overweight range (25.0 to 29.9 kg/m^2^)	46	20.4
Obese range (≥30.0 kg/m^2^)	17	7.6
Education level		
Secondary	69	30.7
University	156	69.3
Socioeconomic status		
Social class of the participant’s family		
Lower class	1	0.4
Working class	14	6.2
Lower-middle class	43	19.1
Middle class	127	56.4
Upper-middle class	38	16.9
Upper class	2	0.9
Having a job (either part-time or full-time)		
Yes	163	72.4
No	62	27.6
Number of people residing in participant’s family of origin household		
1	39	17.3
2–3	111	49.3
4–5	66	29.3
6 or more	9	4.0
Eating habits		
Being currently on a diet		
Yes	31	13.8
No	194	86.2
Considering oneself a healthy eater		
Very slightly or not at all	7	3.1
Somewhat	25	11.1
Moderately so	105	46.7
Mostly	80	35.6
Definitely	8	3.6

**Table 4:** Mean, standard deviation, minimum, maximum, skewness, and kurtosis values of anxiety, cognitive instability, and unhealthy eating for adult women (N = 225).

Measure	Mean	SD	Min	Max	Skewness		Kurtosis	
					Statistic	Std. Error	Statistic	Std. Error
Trait anxiety	43.32	8.66	23	71	0.36	0.16	−0.05	0.32
Cognitive instability	6.41	1.88	3	12	1.88	0.16	−0.27	0.32
Unhealthy eating	6.78	2.49	2	16	0.57	0.16	0.71	0.32

Note: SD: standard deviation; Min: minimum: Max: maximum; Std. Error: standard error.

**Table 5:** Principal components analysis: reduction of the dimensionality of the data to a small number of components.

		Component			
Total Variance Explained		1	2	3	4
Initial eigenvalues	Total	3.193	1.467	1.333	1.013
	% of variance	29.032	13.338	12.117	9.206
	Cumulative %	29.032	42.369	54.487	63.692
Extraction sums of squared loadings	Total	3.193	1.467	1.333	1.013
	% of variance	29.032	13.338	12.117	9.206
	Cumulative %	29.032	42.369	54.487	63.692
Rotation sums of squared loadings	Total	2.463	1.871	1.490	1.038
	% of variance	22.391	17.011	13.543	9.432
	Cumulative %	22.391	39.402	52.945	62.377

**Table 6:** Extraction of four principal components from the FFQ: rotated component matrix ^a^.

Individual FFQ Question: During the Past 2 Months How Often Have You Eaten or Drunk ….?	Component			
	1	2	3	4
Fruit	0.878			
Vegetables	0.729			
Whole grains	0.726			
Fresh foods	0.697			
Gluten-free foods		0.778		
Low sodium foods		0.764		
Organic foods		0.720		
Something sweet and unhealthy (i.e., candy or cake)			0.853	
Snack food			0.842	
Water				0.954

Extraction method: principal components analysis. Rotation method: varimax with Kaiser normalization. ^a^ Rotation converged in 4 iterations. The size of PC loadings was lower than 0.4.

**Table 7:** Mediation analysis: the effect of cognitive instability in the relationship between trait anxiety and unhealthy eating in adult women.

Variable/Effect	*b*	SE	*t*	*p*	95% Confidence Interval	
Trait anxiety → Unhealthy eating	0.010	0.024	−0.430	0.667	−0.059	0.038
Trait anxiety → Cognitive instability	0.139	0.011	12.496	*p* < 0.001	0.117	0.161
Trait anxiety → Cognitive instability → Unhealthy eating	0.244	0.113	−2.153	0.032	−0.468	−0.020
Effects						
Direct	0.010	0.024	−0.430	0.667	−0.059	0.038
Indirect	0.034	0.016			−0.065	−0.002
Total	0.044	0.019	−2.345	0.019	−0.082	−0.007

## Data Availability

The dataset used during the current study is available from the corresponding author upon reasonable request.
